# 2020 vision or myopia? A personal perspective on the future of cancer imaging and an introduction to the sequels to the “How I Read Series”

**DOI:** 10.1186/s40644-019-0194-8

**Published:** 2019-02-06

**Authors:** Rodney J. Hicks

**Affiliations:** The Sir Peter MacCallum Department of Oncology, the University of Melbourne, Level 5, VCCC Building, 305 Grattan St, Melbourne, VIC 3000 Australia

Almost a decade ago, a French cancer centre, for its 10th anniversary celebratory magazine, asked me to write a brief prediction of how oncology might look in 2020. I wrote (loosely translated from the original French version);

*“Over the next ten years, I see a “seismic” shift in how new cancer treatments will be developed and evaluated in clinical trials. I believe that randomized trials that seek to minimize the effects of disease heterogeneity will be recognized as not being able to lead to significant advances in the treatment of cancer patients. Instead, there will be a movement toward smaller trials in targeted patient groups. These groups will be selected or at least enriched with the patients most likely to benefit from a particular treatment because of the expression of a potential therapeutic target. These targets will be identified by techniques such as genomic analysis that is increasingly rapid and affordable thanks to a new generation of sequencers or more specific molecular imaging techniques. Similarly, the efficiency of the treatments will be established by the early demonstration of modulation of the target and by highlighting the cascading effects of this modulation of the target. As data accumulates on cellular events and when cellular biomarkers, serum proteomic signatures or molecular imaging signatures demonstrate that they are valid predictors of response and influence both the duration and quality of life, we will progressively move towards a molecular medicine that is personalized, adapted, informed and guided by these new techniques. This medicine will leave the domain of research to be used in every day clinical practice.”*

There is an iconic Australian movie entitled *The Castle.* Many of its classic lines have entered into the Australian vernacular. One of them pertains to the likelihood of an improbable event. It is; “*He’s dreaming*”. When I wrote the forecast above, I was clearly dreaming. Although elements of my predictions are closer, it is a future that still hasn’t been realized in large measure. As we approach the end of the second decade of the twenty-first century it is pertinent to look back on the momentous changes that have occurred in the practice of oncology since the beginning of this millennium and to contemplate the changes that might still be to come in the field and how they might impact the practice of cancer imaging.

Although the direct clinical impact of the revolution in genomic analysis of cancers, exemplified by The Cancer Genome Atlas (TCGA), has been questioned [1], there can be no doubt that understanding of the biological basis of cancer has improved immeasurably as a result. Thousands of articles describing the key mutations in a wide array of malignancies have been published. Over the past 2 decades, most of the key oncogenic pathways have been intimately detailed. Small molecule inhibitors of aberrant gene expression have provided effective treatments for a number of cancers that previously lacked therapeutic options once they had become metastatic. These have included treatments both for rather indolent tumours, like gastrointestinal stromal tumour (GIST), and for aggressive malignancies, like melanoma. Despite the effectiveness of drugs like imatinib in GIST [2] and vemurafenib in melanoma [3, 4], not all cells respond to treatment and development of resistance is almost universal after a time. Molecular imaging played a key role in identifying that rapid changes occur in glycolytic metabolism with successful abrogation of signaling driven by oncogenes including mutant cKit in GIST [5] and vemurafenib in BRAF-V600E melanoma [6]. This, in turn, led to further studies to elucidate the mechanisms of metabolic reprogramming and mechanisms of resistance [7].

Beyond individual gene mutations, a “systems biology” approach has identified hallmarks of cancer that enable cancers to develop and which can be exploited as therapeutic targets [8]. For example, the ability of some cancers to generate neovasculature has been countered by the development of anti-angiogenic therapies. However, again, primary refractory disease or secondary resistance are common. The fundamental importance of gene signaling pathways to normal cellular homeostasis means that many of the therapeutic approaches also perturb normal tissues and lead to significant side-effect profiles. Thus, despite the great enthusiasm that greeted the first wave of targeted therapies that ushered in the twenty-first century, these treatments are now regarded as primarily agents to delay progression of disease rather than to cure it.

While it has long been thought, and more recently proven, that the immune system plays a role in cancer, the last decade, in particular, has led to an acceleration in the understanding of the complex interactions between cancer cells and an array of cells involved in the immune response to malignant transformation [9]. As a consequence, novel therapeutic agents have been developed that modulate the immune response, especially so-called immune check-points that suppress recognition or killing of cancer cells. Although not all cancers respond to such agents, dramatic responses and some apparent cures are well-documented albeit with a significant associated immune-related toxicity profile [10]. Thus, this millennium has seen the addition of targeted therapy and immunotherapy to the traditional therapeutic pillars of surgery, radiation (external and internal) and chemotherapy.

With the increasing complexity of therapeutic options and the development of new diagnostic approaches including increasing access to molecular pathology, gene panels, circulating tumour cells and cell-free DNA, multidisciplinary care has become ever more important and has seen definition of clinical pathways for individual patients being determined by consensus of clinicians from several specialties rather than by individual practitioners. The multi-disciplinary team now includes pathologists and imaging specialists as core participants. This has been one of the major changes in oncology practice over the past two decades.

Imaging of cancer has long played a role in defining the local and regional extent of cancer in order to evaluate the ability of surgery or radiotherapy to effect cure and to exclude the presence of metastatic disease that would generally preclude cure in most individuals. In cases with systemic spread, the primary role of imaging has been to provide a baseline against which to assess therapeutic response. While these functions remain of fundamental importance in modern oncology, there is increasing recognition that imaging will be vital in characterizing disease, particularly with respect to susceptibility to novel therapeutic approaches. For example, contrast-enhancement patterns may be more important as a biomarker for response to anti-angiogenic therapy than as a method to increase the sensitivity for detection of disease. It may also play a critical role in the evaluation of side-effects of immunotherapy in parallel with evaluation of therapeutic response [11].

In an era of rapidly evolving science within oncology, imaging specialists need to keep abreast of advances in other diagnostic domains as well as driving or adopting advances in their own field. If imaging is to be integrated into modern precision medicine as a key platform technology, we need to embrace change. To address these issues, we have commissioned articles that provide a sequel to the successful “How I Read” series of articles, which detailed how experts in the field approach many of the day-to-day practical issues of scan acquisition and interpretation [12]. These articles will review four exciting areas of change in imaging. These will be; instrumentation advances, innovation in imaging agents, informatic approaches to data analysis and investigational strategies for imaging trials. As children we used to call people who wore glasses, “four-eyes”! This 4-Is series will hopefully provide us a clear vision for cancer imaging beyond 2020.

The review on instrumentation will report advances in equipment and image processing, which will enable more detailed analysis of tissue characteristics. New detector technologies, including digital photon detectors, whole-body scanning techniques for PET and MRI and the evolution of hybrid imaging devices will be of particular interest. A paper covering innovation in imaging agents will discuss how chemists are leveraging of advances in basic science to develop new companion diagnostics that will be applicable to selection and monitoring of targeted and immune therapies. These will include radiotracers, MRI agents and optical imaging. The review on informatics will detail the methods used for the extraction and analysis of data from imaging studies. The topics of radiomics and artificial intelligence through machine-learning will be important aspects of this paper. The final review will describe investigational strategies for validating the impact of these advances on patient care and will be critical to improve the chances that they will be both adopted and appropriately funded. Clinical trial design in imaging has long been neglected and has often led to poor quality data, which, in turn, has impeded efforts to make new techniques available to patients by establishing the evidence-base for reimbursement.

As co-Editors-in-Chief and practicing cancer imaging specialists, my colleague, Annick D. Van den Abbeele, MD and I believe that we live in exciting times for the cancer imaging specialist and despite the challenges posed by rapid technological development, we are likely to play an ever-increasing role in the selection and monitoring of cancer therapies and thereby contribute to improved patient outcomes.

## Abbreviation

DNA: Deoxyribonucleic acid
